# Variations of Serum Oxidative Stress Biomarkers under First-Line Antituberculosis Treatment: A Pilot Study

**DOI:** 10.3390/jpm11020112

**Published:** 2021-02-09

**Authors:** Andreea-Daniela Meca, Adina Turcu-Stiolica, Elena Camelia Stanciulescu, Ana Marina Andrei, Floarea Mimi Nitu, Ileana Monica Banita, Marius Matei, Catalina-Gabriela Pisoschi

**Affiliations:** 1 Department of Pharmacology, University of Medicine and Pharmacy of Craiova, 200349 Craiova, Romania; andreea_mdc@yahoo.com; 2 Department of Pharmacoeconomics, University of Medicine and Pharmacy of Craiova, 200349 Craiova, Romania; 3 Department of Biochemistry, University of Medicine and Pharmacy of Craiova, 200349 Craiova, Romania; camiparsot@yahoo.com (E.C.S.); marinafusaru@yahoo.com (A.M.A.); c_pisoschi@yahoo.com (C.-G.P.); 4 Department of Pneumology, University of Medicine and Pharmacy of Craiova, 200349 Craiova, Romania; dr_nitumimi@yahoo.com; 5 Department of Histology, University of Medicine and Pharmacy of Craiova, 200349 Craiova, Romania; monica.banita@yahoo.com (I.M.B.); mariusmatei44@yahoo.com (M.M.)

**Keywords:** oxidative stress, tuberculosis, glutathione, superoxide-dismutase, catalase, antioxidant

## Abstract

Tuberculosis (TB) is one of the highest infectious burdens worldwide, and pathogenesis is yet incompletely elucidated. Bacilli dissemination is due to poor antioxidant defense mechanisms and intensified oxidative stress. There are few recent studies that analyzed and compared free radicals or antioxidant status before and after anti-TB treatment. Hence, the present study underlines the need to identify oxidative stress as it could be a useful tool in TB monitorisation. Thirty newly diagnosed patients with pulmonary TB were included after signing an informed consent. Blood was collected before receiving first-line anti-tubercular therapy (T0) and after 60 days (T2). Spectrophotometric methods were used to quantify oxidative parameters (TBARS—thiobarbituric acid reactive species); enzymatic antioxidants such as SOD (superoxide dismutase), CAT (catalase), GPx (glutathione peroxidase), and TAC (total antioxidant capacity); and non-enzymatic antioxidants such as GSH (reduced glutathione). A moderate positive correlation was found between GSH and TAC (*r* = 0.63, *p*-value = 0.046) and GSH and SOD (*r* = 0.64, *p*-value = 0.041) at T2. Increased values of GSH, CAT, and SOD were noted at T2 in comparison with T0, while GPx, TAC, and TBARS decreased at T2. A better monitorisation in TB could be based on oxidative stress and antioxidant status. Nevertheless, restoring redox host balance could reduce TB progression.

## 1. Introduction

The World Health Organisation (WHO) announced in 2019 that, in Europe, 30 people are diagnosed with active tuberculosis (TB) every hour [1]. Although TB is still one of the highest burdens worldwide, pathogenic mechanisms are yet incompletely known [2,3,4,5]. Identification of oxidative stress involvement in TB could lead to better disease monitorisation [6].

*Mycobacterium tuberculosis (M.tb)*, the aetiological agent of TB, is capable of high and long-term persistence within macrophages by inhibiting phago-lysosomal fusion [3,4,5]. Latent infection appears as a response of *M.tb.* maintenance in a low replicative state (latent TB), with support of T- and B-lymphocytes [3,4,5]. If alveolar macrophages further attract more immune cells such as monocytes and neutrophils, an inflammatory granuloma will be formed, as *M.tb*. subverts host immunity and survives [7,8]. Although, within the granuloma, immune cells interact and trigger immune responses in order to control TB, macrophages can undergo respiratory burst, generating oxidative stress and initiating active pulmonary TB [4,7,8]. Bacilli release and infection progression are consequences of lipid peroxidation, parenchymal tissue destruction, and insufficient antioxidant defence mechanisms [3,9,10]. The higher the bacterial load, the more advanced will be the necrosis; therefore, an immunological fight between *M.tb.* and the individual will establish disease consequences [3,9,11,12].

Nevertheless, host damage is generated owing to the imbalance between reactive oxygen species (ROS) and antioxidants [10,12]. ROS reduce defence autophagic and apoptotic host mechanisms, but can also inhibit the pathogen development along the lung, by destroying mycobacterial DNA, activating lipid peroxidation, and destabilizing proteins [5,7,10,12,13]. Moderate ROS concentrations ensure functionality of signalling pathways and immunoregulation, but an excess affects cellular homeostasis in an uncontrolled manner [14,15]. Lipid peroxidation leads to *M.tb.* membrane disruption by affecting the double chemical bonds of polyunsaturated lipids [5]. Even more highly toxic and powerful free radicals, such as superoxide (O_2_^-^) and hydroxyl (OH^-^), are generated after mitochondrial oxidative burst [16]. However, *M.tb.* antioxidant system could cross over ROS activity and lead to long-term bacilli survival and inflammatory response [5].

On the other hand, the incremental autophagic capacity of macrophages can be supported through host antioxidant activity [5]. Both enzymatic (superoxide dismutase (SOD), catalase (CAT), and glutathione peroxidase (GPx)) and non-enzymatic antioxidants (reduced glutathione (GSH)) remove ROS before DNA, proteins, or lipids attack and ensure direct anti-inflammatory effects and adaptative immunological response [5,15]. Superoxide radicals can be converted through SOD intervention to H_2_O_2_, as an adaptive response [6,15]. CAT counteracts H_2_O_2_ higher concentrations, while GPx catalyses reduction of both organic peroxides and H_2_O_2_ [14,15]. GPx, glutathione oxidase, and glutathione reductase provide cellular detoxification and lower ROS damage through the glutathione redox cycle, using the sulfhydryl group of cysteine as a proton donor [15]. Therefore, antioxidants prevent severe TB manifestations and counteract tissue degradation [5,15].

The amount of ROS generated to kill *M.tb.* can be spectrophotometrically measured by following plasmatic levels of malondialdehyde (MDA) and other thiobarbituric acid reactive species (TBARS) (conclusive for lipid peroxidation) or protein carbonyls thiols (conclusive for protein oxidation) [8,12,17]. Enzymatic antioxidants can also be traced and quantified in order to measure the host capacity to prevent any oxidative damage [8,12,17].

Our pilot study compared blood levels of oxidative and antioxidant biomarkers in pulmonary TB patients, before and after 60 days of first-line antituberculosis treatment, and further correlated the values, as awareness of oxidative stress in pulmonary TB could lead to better understanding of TB pathogenesis and possible monitorisation of this highly infectious disease.

## 2. Materials and Methods

### 2.1. Study Design

The prospective study received approval from the Ethics Committee from The University of Medicine and Pharmacy of Craiova, Romania (Nr.5/17.01.2019). We included thirty newly diagnosed pulmonary active TB adults, of both genders, aged between 18 and 65 years, who had not received more than one week of first-line anti-TB treatment. Patients hospitalized at ”Victor Babeș” Clinical Hospital of Infectious Diseases and Pneumophthisiology Craiova and Pneumology Hospital Leamna, Dolj County, from Romania, were recruited after obtaining their written informed consent. All the patients were diagnosed with active TB after sputum-smear positivity test and further confirmed through radiologic and clinical examinations. Patients diagnosed with other different chronic illness, pregnant women, and children met exclusion criteria of our study. After examination of patient disease history, individuals with extra-pulmonary TB, HIV infection, cancer, hypertension, hepatic or renal insufficiency, diabetes, and parathyroid gland dysfunctionalities were excluded. Patients who were administered antioxidants, vitamin D supplements, or medication that may interfere with vitamin D plasmatic levels (theophylline, some anticonvulsants, thiazide diuretics, corticosteroids) were also excluded, as vitamin D presents immunomodulatory roles and influences oxidative biomarkers [18,19,20].

Venous blood samples were collected before receiving treatment (T0) and after 60 days (T2) of initial intensive phase of first-line regimen (isoniazid, rifampicin, ethambutol, pyrazinamide). TB regimen was given in accordance with the Revised National Tuberculosis Control Program and DOTS strategy (direct observed short course chemotherapy), adjusting doses by patients’ weight. We used heparinized, K_2_EDTA, and without any anticoagulant BD vacutainers (Becton Dickinson, USA), according to the manufacturer’s recommendations, to collect blood from each patient who approved participation. Part of blood samples was further processed and centrifuged at 1000× *g* (Eppendorf centrifuge 5417R, Eppendorf AG, Hamburg, Germany), within 30 min after collection, and then serum/plasma, whole blood, and hemolysate were aliquoted (minimum of 500 µL). All samples were stored at −80 °C until laboratory assessments of biomarkers.

### 2.2. Biochemical Analysis

Oxidative stress biomarkers were quantified using spectrophotometric methods in vitro, by manual use [21,22,23,24,25,26]. TBARS were measured in order to quantify lipid peroxidation. Enzymatic activities of SOD, CAT, GPx, and TAC (total antioxidant capacity) were assessed using hemolysates or whole blood for standard procedures. GSH was the only non-enzymatic antioxidant quantified in the present study.

#### 2.2.1. Determination of SOD Activity

For measurement of SOD activity, the blood was processed for haemolysis. Centrifugation at 1100× *g*, for 10 min, was performed for 0.5 mL of blood and the upper plasma was removed. Erythrocytes pellet was washed four times with physiologic saline solution, centrifuging 10 min after each wash, and after the final wash, erythrocytes were diluted at 2 mL with cold redistilled water, mixed, and kept at +4 °C for 10 min. A 100-fold dilution of the lysate with 0.01 M phosphate buffer saline (PBS) pH = 7 is necessary before performing SOD assay using a Ransod kit (Randox Laboratories, Crumlin, County Antrim, UK). SOD activity was quantified from the degree of inhibition of the reaction between superoxide radicals, generated after the oxidation of xanthine (0.05 mmol/L) with xanthine oxidase (80 UI/L), and 2-(4-iodophenyl)-3-(4-nitrophenol)-5-phenyltetrazolium chloride (INT, 0.025 mmol/L). A DU65 UV/VIS spectrophotometer (Beckman, Germany) was used to measure the absorbance of the pink formazan at 505 nm. A unit of SOD activity represents the quantity of SOD that determines a 50% inhibition of INT reduction rate. In order to determine the percentage of inhibition for each sample, diluted samples and standards rates were converted into percentages of a sample diluent rate after subtraction from 100%, and assigned a value for the rate of the uninhibited reaction. SOD activity was expressed as SOD units/mL of whole blood.

#### 2.2.2. Determination of GPx Activity

GPx activity was measured using the Ransel kit (Randox Laboratories, Crumlin, County Antrim, UK) based on a UV method proposed by Paglia and Valentine [27]. GPx from the sample of heparinized whole blood catalyses the oxidation of GSH (4 mmol/L) by cumene hydroperoxide (0.18 mmol/L). Oxidised glutathione (GSSG) is converted to GSH in the reaction catalysed by glutathione reductase (>0.5 U/L) in the presence of NADPH (reduced form of nicotinamide adenine dinucleotide phosphate) (0.34 mmol/L), leading to a decrease in absorbance, which was further measured at 340 nm. The optical density was measured using the UV/VIS spectrophotometer Beckman. Heparinized whole blood was previously diluted with diluting agent and Drabkins’ reagent to inhibit the interference of other blood peroxidases. GPx activity was expressed as U/L of haemolysate.

In order to determine GSH concentration and CAT activity, we blended equal volumes of blood and cold distillate water, and then centrifugated the mixture for 15 min at 4000× *g* (Eppendorf refrigerated centrifuge 5417 R, Eppendorf AG, Hamburg, Germany) to obtain a clear haemolysate.

#### 2.2.3. Determination of CAT Activity

For CAT activity determination, we used the Aebi method based on direct quantification of hydrogen peroxide decomposition rate by CAT and haemoglobin within the sample [28,29,30]. All the samples were diluted 1:10, mixed with 0.07 M PBS, pH = 7, following an incubation at 37 °C for 10 min. The decrease of absorbance at 240 nm after hydrogen peroxide addition was read using the DU65 UV/VIS Beckman spectrophotometer. CAT activity was assessed through the molar extinction coefficient of hydrogen peroxide, as one unit of CAT is considered to decompose 1 μmol of hydrogen peroxide during 1 min. Therefore, CAT activity was expressed as unit per mg of haemoglobin (U/mgHb), measured using flow-cytometry on automated analyzers.

#### 2.2.4. Determination of GSH Concentration

Samples of each haemolysate were treated with 5% trichloroacetic acid (TCA, *v/v*), blended, and separated after 5 min of centrifugation at 28,000× *g* at a lower temperature (4 °C). Each supernatant was then combined with a solution of 5,5’-dithio-bis-(2-nitrobenzoic acid) (DTNB, Ellman’s reagent) (0.01 M) in 0.07 M PBS, pH = 8 (1:50 ratio, *v/v*), and incubated for 45 min in the dark and at room temperature (RT). The absorbance of the pale-yellow product at 412 nm was measured with a UV/VIS spectrophotometer Kruss (Hamburg, Germany) and then converted to GSH concentration according to the GSH standard curve and expressed as mg/dL.

#### 2.2.5. Total Antioxidant Capacity (TAC) Assay

A spectrophotometrically evaluation of TAC in blood samples is one of the assays usually performed to assess the body’s ability to produce antioxidants and combat the oxidative stress [21,22,23,24,25,26]. Each plasma sample was diluted with 0.01 M PBS, pH = 7.4 (1:25 ratio, *v/v*), combined in equal volumes with 0.1 mM 2,2′-diphenyl-1-picrylhydrazyl radical reagent (DPPH), and further incubated in the dark and at RT for 30 min. After a 3 min centrifugation at 20,000× *g*, the absorbance at 520 nm was read with the UV/VIS spectrophotometer Kruss. All the reagents were provided by Sigma-Aldrich, Germany and TAC was expressed as mmol DPPH/L.

#### 2.2.6. Thiobarbituric Acid Reactive Substances Assay

This assay was performed for the evaluation of the lipid peroxidation level by quantifying plasmatic MDA concentration, its major final product [21,22]. Plasma samples were mixed with equal volumes of 5% TCA and Tris-HCl (0.2 M) pH = 4.7 (*v/v*) and incubated 10 min at RT. Next, 0.55 M thiobarbituric acid (TBA) solution in sodium sulphate (2 M) was added and the mixture was incubated at 90 °C for 45 min [24,25]. After ice cooling, a 3 min centrifugation (15,000× *g*) was carried out with the same refrigerated centrifuge. During this procedure, a coloured product between MDA and TBA is formed, with its absorbance at 532 nm being measured with the same UV/VIS spectrophotometer Kruss. The molar extinction coefficient of MDA (1.55 × 105 M^−1^cm^−1^) supported our calculation of TBARS concentration, expressed as μmol/L. Almost all the reagents were provided from Sigma-Aldrich, Germany, except TBA, obtained from Fluka.

### 2.3. Statistical Analysis

Data were analyzed using the GraphPad Prism 9.0 software (GraphPad Software, San Diego, CA, USA). The results are presented as mean ± standard deviation (SD) and range. The nonparametric Wilcoxon matched-pairs signed rank test was used to evaluate the significant differences between variables at T0 and T2. It was considered statistically significant at the 5% level (two-tailed).

The strength of the quantitative relationship between the different clinical characteristics of the patients was measured with Spearman coefficients and heatmap of correlation matrix.

## 3. Results

We included 30 patients, but only 15 were analyzed during the period of study, as for them, it was possible to concomitantly quantify all the parameters at T0 and T2. Their range for age was 32–61 years and for weight was 39–64 kg. Table 1 includes the demographic characteristics of our patients, whereas Table 2 summarizes the clinical characteristics at both points in time.

CAT values were significantly different between the two periods of time (before and after the TB treatment). CAT activity was lower at baseline than after treatment, as shown in Table 2. 

As Table 2 displays, for the other biomarkers, we did not find significant differences between the values obtained before and after the TB treatment.

**Figure 1:**
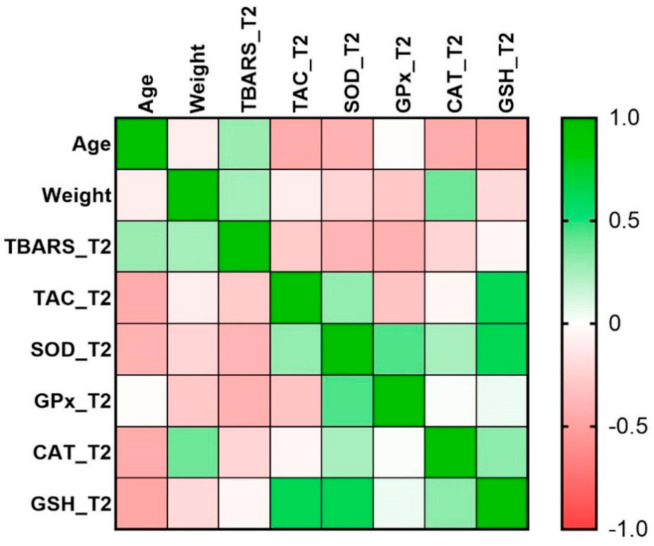
Heatmap of correlation matrix. TBARS, thiobarbituric acid reactive species; TAC, total antioxidant capacity; SOD, superoxide dismutase; GPx, glutathione peroxidase; CAT, catalase; GSH, reduced glutathione. T2, after 60 days of antituberculosis treatment.

We found a moderate positive correlation between GSH and TAC (*r* = 0.63, *p*-value = 0.046) and GSH and SOD (*r* = 0.64, *p*-value = 0.041) at T2, as shown in Table 3 and Figure 1.

## 4. Discussion

Our pilot study analysed oxidative stress biomarkers and antioxidants status in pulmonary active TB patients, before and after two months of anti-TB treatment. *M.tb.* generates ROS through mitochondrial respiratory burst and intensively affects both normal lung function and host immune responses [5,6,23]. An altered antioxidant profile further contributes to disease evolution owing to an incapacity to properly remove the oxidative burden [5,6,23]. Superoxide radicals and hydrogen peroxide are products of flavoenzymes; cyclo- and lipo-oxygenases; as well as NADPH-dependent oxidase, a specific enzyme of phagocytes [23]. Hydrogen peroxide represents an important intermediate in more reactive radicals’ synthesis, such as the hydroxyl radical (obtained through Fenton reaction, catalysed by Fe^2+^ or Cu^2+^), as the only ROS that penetrates cellular membranes [23]. CAT and GPx are responsible for hydrogen peroxide removal, while SOD counteracts superoxide radicals by dismutation to hydrogen peroxide [5,6,23]. However, if these enzymes are insufficient, free radicals accumulate within pulmonary tissue and attack membrane lipids, leading to lipid peroxidation and MDA synthesis as one of its final products [23].

The results we obtained underline higher TBARS plasmatic values and lower antioxidant defence at T0, suggesting increased lipid peroxidation rates, dysfunction of redox homeostasis, cytotoxic consequences, and implication of increased ROS in TB pathogenesis [12,13,23]. Nevertheless, ROS are used in *M.tb.* recognition and initiation of immune response [14]. ROS formation is dependent on proinflammatory cytokine and chemokine activity, which leads to a vicious circle in TB, as the bacilli chemoattract different immune cells and maintain tissue remodelling [14]. Decreased lipid peroxidation at T2, assessed through TBARS, suggests a delay or even prevention of pulmonary fibrosis, also due to higher plasmatic values of CAT and SOD that act as ROS scavengers [12,13]. Rajopadhye et al. [12] obtained similar results with our pilot study, as they correlate TBARS increased values with TB severity and pulmonary fibrosis development [12]. Lower TBARS values also emphasize a lower lipid peroxidation rate, suggesting a decrease in oxidative stress [23] after two months of DOTS.

All patients included in the study suggested poor immunity as they were diagnosed with malnutrition, also confirmed by statistical analysis regarding demographic and clinical data (Table 1, Table 2). The incapacity of neutralising oxidative stress, due to lower weight and wasting, is one of the most important risk factors in developing active TB [7,10,31,32]. Malnutrition and deficiency in various immunomodulatory vitamins, especially vitamin D deficiency, lead to weak promotion of monocytes and T-cells maturation, increased proinflammatory cytokine activity, and postponed phagosome-lysosome fusion by interfering with gene transcription [31,32,33].

Enzymatic antioxidants are present in higher concentrations in erythrocytes, and were thus quantified using hemolysates. Cellular oxidative damage, loss of mitochondrial membrane integrity, and impaired cellular function can also be controlled through glutathione activity, which regulates the redox cycle and maintains antioxidant functionality of ascorbic acid and tocopherol [8,14,15,16]. After 60 days of first-line treatment, GSH values increased, with its possible involvement in modulating cytokine activity and host response [17].

Although there are no aliments known until now with a direct antimycobacterial effect, proper body mass index can ensure host defence mechanisms, by upregulating intracellular GSH biosynthesis, one of the most important antioxidants [17]. Imbalance between ROS and antioxidants, due to intense and chronic macrophage activation [14], implies inadequate removal of oxidative stress, thus affecting the membrane and DNA of pulmonary host cells through an increased rate of lipid peroxidation. Our results agree with the findings in previous studies [10,33,34]. Isoniazid, one of the most administered first-line antituberculotic drugs, stimulates oxidative stress biomarkers and increases hepatotoxicity and nephrotoxicity, concomitant with antioxidant status reduction (SOD, GSH, and TAC), as Hassan et al. and Sharma et al. underline using rat models [33,34]. Needless to mention that all of our patients received first-line antituberculotic treatment.

SOD is involved in transforming superoxide radicals in less reactive peroxide, which is further reduced by CAT to oxygen and water, thus providing tissular protection [13,14]. Furthermore, ROS are eliminated through GPx and glutathione reductase activity, based on conversion of cellular GSH to its oxidized form and regeneration [13,14]. In fact, GPx is abundant in parenchymal lung cells, suggesting its particular implications in patients diagnosed with pulmonary active TB [35]. GSH is used in hydrogen peroxide reduction catalysed by GPx [23]. The reaction involves oxidation of Se- to SeOH, which further adds one to two sulfidic radicals, regenerating to Se- [23]. A significant reduction of SOD and CAT has been noted at T0 for our patients in comparison with T2, similar to other studies [10,12,33,34]. On the other hand, even though GPx and TAC slightly decreased at T2, it might be due to the small number of individuals who gave consent in participating in our study and a short timeline of monitorisation.

One of the strengths of our study is represented by the analysis of oxidative stress biomarkers and antioxidants status at two points of time. An evaluation of lipid peroxidation could lead to a better understanding of TB pathogenesis and could represent a useful tool in identification of individuals exposed to higher risk of progression [23]. To the best of our knowledge, there are few available recent studies that reported these plasmatic changes by comparing their immunomodulatory roles [10,12,33,34]. Moreover, we identified few studies that reported changes in plasmatic investigations before and after anti-TB treatment in humans [10,12]. We found that only Vidhya et al. analysed oxidative biomarkers status in pulmonary TB patients before and after pharmacotherapy, in the last 5 years, and they suggested that antioxidant co-supplementation could be useful in reducing TB severity [10]. All the patients included were only diagnosed with pulmonary active TB, which represents another strength, as comorbidities could influence variation of oxidative stress biochemical parameters. We also quantified alternative biomarkers specific for oxidative stress, instead of MDA, because of their higher stability and specificity and lower rate of variation through analysis steps [36].

The limitations of our study include the small number of patients, as it has been difficult to find new patients only diagnosed with pulmonary TB. Even further, our study was conducted between 1 November 2019 until 1 April 2020, when the COVID-19 pandemic forced us to stop visiting the two hospitals and including more TB patients. It might have also been interesting to compare our results from TB patients’ samples with a control group, not diagnosed with pulmonary TB, to highlight even more the oxidative damage in case of mycobacterial infection. A follow-up study assessing oxidative stress and antioxidants status in TB patients, after six months of antimycobacterial treatment, would also be helpful in establishing TB management strategies.

## 5. Conclusions

The present pilot study underlines the involvement of increased oxidative species and reduction of antioxidant status in developing pulmonary active TB. A host attempt to decrease lung injury by restoring prooxidant/antioxidant balance has also been noted in active pulmonary TB patients, possible leading to increased immunomodulatory effects.

All in all, the pathological outcomes are decided by a tremendous fight between antioxidants and pro-oxidants. Possible nutritional supplementation, based on antioxidants, could represent a novel approach in controlling TB. Further studies regarding modulation of redox balance need to follow up on biochemical changes in order to properly manage this public threat.

## Figures and Tables

**Table 1:** Demographic characteristics of the patients.

Demographic Data	Values: Mean (±SD) or *n* (%)
Age	48.80 (±9.283)
Weight	54.27 (±8.189)
Gender	
Female	4 (26.7%)
Male	11 (73.3%)
Environment	
Urban	4 (26.7%)
Rural	11 (73.3%)

**Table 2:** Biochemical characteristics of the patients.

Biomarkers	T0 Mean ± Standard Deviation (range)	T2 Mean ± Standard Deviation (range)	*p*-Value
TBARS (μmol/L)	0.73 ± 0.29 (0.50–1.36)	0.68 ± 0.29 (0.39–1.23)	0.551
TAC (mmol DPPH/L)	49.49 ± 4.94 (38.15–56.23)	49.14 ± 9.80 (29.33–63.51)	0.691
SOD (U/mL)	283.99 ± 16.05 (231.00–297.15)	291.62 ± 8.03 (275.62–305.66)	0.074
GPx (U/L)	1617.35 ± 750.40 (656.14–3768.58)	1441.82 ± 345.52 (1093.56–2187.12)	0.333
CAT (U/mgHb)	1.07 ± 0.63 (0.41–2.38)	1.41 ± 0.77 (0.62–3.54)	0.008 *
GSH (mg/dL)	7.63 ± 1.60 (4.80–10.60)	8.16 ± 3.01 (4.00–12.60)	0.490

* *p* < 0.05, significantly different. TBARS, thiobarbituric acid reactive species; TAC, total antioxidant capacity; SOD, superoxide dismutase; GPx, glutathione peroxidase; CAT, catalase; GSH, reduced glutathione; DPPH, 2,2′-diphenyl-1-picrylhydrazyl radical reagent.

**Table 3:** Spearman coefficients with correlation matrix.

	Age	Weight	TBARS_T2	TAC _T2	SOD _T2	GPx _T2	CAT _T2	GSH_T2
**Age**	1.00							
**Weight**	−0.10	1.00						
**TBARS_T2**	0.29	0.26	1.00					
**TAC_T2**	−0.45	−0.10	−0.28	1.00				
**SOD_T2**	−0.40	−0.22	−0.40	0.29	1.00			
**GPx_T2**	−0.02	−0.29	−0.42	−0.32	0.45	1.00		
**CAT_T2**	−0.45	0.39	−0.23	−0.05	0.24	0.02	1.00	
**GSH_T2**	−0.47	−0.20	−0.05	0.63 *	0.64 *	0.05	0.32	1.00

* *p* < 0.05. TBARS, thiobarbituric acid reactive species; TAC, total antioxidant capacity; SOD, superoxide dismutase; GPx, glutathione peroxidase; CAT, catalase; GSH, reduced glutathione.

## Data Availability

The data presented in this study are available on request from the corresponding author. The data are not publicly available due to patients’ confidentiality.
